# Factors Affecting the Severity of Placental Abruption in Pregnant Vehicle Drivers: Analysis with a Novel Finite Element Model

**DOI:** 10.3390/healthcare10010027

**Published:** 2021-12-24

**Authors:** Katsunori Tanaka, Yasuki Motozawa, Kentaro Takahashi, Tetsuo Maki, Masahito Hitosugi

**Affiliations:** 1 Department of Legal Medicine, Shiga University of Medical Science, Otsu 520-2192, Japan; kttanaka@belle.shiga-med.ac.jp (K.T.); motozawa@ucatv.ne.jp (Y.M.); 2 Hino Memorial Hospital, Hino 529-1642, Japan; taka27@belle.shiga-med.ac.jp; 3 Department of Mechanical Engineering, Tokyo City University, Tokyo 158-8557, Japan; tmaki@tcu.ac.jp

**Keywords:** pregnant women, motor vehicle collision, placental abruption, numerical simulation, finite element model

## Abstract

We clarified factors affecting the severity of placental abruption in motor vehicle collisions by quantitively analyzing the area of placental abruption in a numerical simulation of an unrestrained pregnant vehicle driver at collision velocities of 3 and 6 m/s. For the simulation, we constructed a novel finite element model of a small 30-week pregnant woman, which was validated anthropometrically using computed tomography data and biomechanically using previous examinations of post-mortem human subjects. In the simulation, stress in the elements of the utero–placental interface was computed, and those elements exceeding a failure criterion were considered to be abrupted. It was found that a doubling of the collision velocity increased the area of placental abruption 10-fold, and the abruption area was approximately 20% for a collision velocity of 6 m/s, which is lower than the speed limit for general roads. This result implies that even low-speed vehicle collisions have negative maternal and fetal outcomes owing to placental abruption without a seatbelt restraint. Additionally, contact to the abdomen, 30 mm below the umbilicus, led to a larger placental abruption area than contact at the umbilicus level when the placenta was located at the uterus fundus. The results support that a reduction in the collision speed and seatbelt restraint at a suitable position are important to decrease the placental abruption area and therefore protect a pregnant woman and her fetus in a motor vehicle collision.

## 1. Introduction

Road traffic injuries are a major public health issue. According to the World Health Organization, 1.35 million people die, and 50 million people are injured annually in road traffic collisions worldwide [1]. Therefore, decreasing the number of casualties in road traffic collisions is a priority. Additionally, declining birth rates have recently become a concern, especially in developed countries [2]. There is thus a need to better protect pregnant women and their fetuses from unintentional injuries. A recent systematic review of the injuries of pregnant women suggested that motor vehicle collisions (MVCs) are the leading cause of both maternal and fetal mortality [3]. In the United States, approximately 160 pregnant women are killed annually in MVCs and, if the mother survives, 800–2800 fetuses are killed annually [4,5]. A recent questionnaire survey revealed that 2.9% of pregnant women were involved in MVCs in Japan [6]. Thus, understanding the mechanisms of injury of pregnant women experiencing fetal death in MVCs is important to improve the safety of pregnant women and their fetuses.

Various studies based on real-world MVCs involving pregnant women have contributed to the improvement of maternal and fetal safety [7,8,9,10,11,12]. Although these studies have provided valuable information on the collision situation, drivers’ characteristics, and injuries suffered by pregnant women and their fetuses, there has been a lack of investigation of the biomechanical features of injury mechanisms and the detailed kinematics of vehicle and passenger. To address this issue, mechanical models (dummy models) specific to pregnant women have been developed for the analysis of collisions involving pregnant women [13,14]. A series of front and rear impact sled tests using anthropometric models of pregnant women have been performed to clarify the mechanisms of injuries in pregnant drivers [14,15]. In frontal impacts, the unrestrained pregnant dummy moved forward, and the dummy’s abdomen directly hit the lower rim of the steering wheel even at a low collision speed of 13 km/h; in rear impacts, a similar phenomenon was observed following a rebound even at a low collision speed of 24 km/h. Subsequently, higher abdominal pressure (indicating higher intra-uterine pressure) was obtained with a pregnant woman dummy even for low-velocity impacts. Additionally, it was biomechanically confirmed that wearing a three-point seatbelt reduced the abdominal pressure and prevented contact with the steering wheel in frontal and rear collisions [14].

Placental abruption, which is the premature separation of the placenta from the uterus, accounts for 50–70% of fetal losses in MVCs [16]. Owing to forces transmitted to the uterus on impact, there is a shearing effect between the placenta and the uterus, with deformation of the uterus. An in-depth understanding of negative fetal outcomes thus requires more detailed analysis of the changes occurring in the uterus. Recently, mathematical models have been used in the study of maternal and fetal safety in MVCs. The finite element (FE) approach, which has been adopted widely in the mathematical analysis of artificial structures, such as automotive vehicles, helps us visualize and understand not only biomechanics but also molecular biology [17,18,19,20]. This approach allows a thorough investigation of the mechanisms of a negative fetal outcome, especially placental abruption. Rupp et al. performed simplified computer modeling to determine the most likely cause of placental abruption from impact loading and found that pressure gradients generated by the inertia of the amniotic fluid and strains in the uteroplacental interface were the dominant mechanisms of placental abruption [13]. They also found that placental abruption occurred when the strain in the uteroplacental interface exceeded 60%. Moorcroft et al. simulated various conditions of MVCs using similar methods and concluded that the peak uterine strain determined by the model was well correlated with the risk of fetal death [21]. Auriault et al. developed a model of a pregnant woman and determined the adverse fetal outcome predictor to be the intra-uterine pressure at the amnion on the placenta in the simulation of MVCs involving pregnant women [22,23]. They therefore considered shear stress, uterine pressure, and the changing rates of these tissues as indices of a negative fetal outcome.

However, the severity of placental abruption depends on the area of uterus–placenta separation. The percentage of separated area has previously been used to estimate the severity of this condition; the severity is classified into three grades of percentage of separated area, i.e., less than 30%, from 30% to 50%, and more than 50%; the percentages of fetal fatalities after vaginal delivery are 30%, 79%, and 100%, respectively [24]. The risk of fetal loss is suspected to increase with the extent of placenta separation [25]. The clinical diagnosis of placental abruption may be difficult if the separated area is small and there are no adverse changes to the pregnant woman or the fetus. Indeed, there have been fetal negative outcomes in cases lacking marked clinical symptoms and even for low-velocity vehicle collisions [8]. In these cases, although a diagnosis was not confirmed, the occurrence of placental abruption was strongly suspected retrospectively. Therefore, determining the threshold for the occurrence of abruptio placenta is inadequate when suspecting a negative fetal outcome. To improve fetal safety in MVCs, it is necessary to identify and diminish the factors that promote the separation leading to placental abruption. Reported computer simulation models can reproduce the uterine strain under given crash conditions, but they do not clarify the extent of placental abruption [16,21,26].

In the present study, we developed a novel mathematical model for evaluating the separated area quantitatively in placental abruption. We used the model to determine the risk factors of a negative fetal outcome.

## 2. Materials and Methods

We considered the situation of fetal loss following a frontal MVC involving a small, unrestrained pregnant woman with a gestational age of 30 weeks, whose abdomen stroke the lower rim of the steering wheel. We first constructed a novel FE model of the pregnant woman for estimating the area of placenta abruption. The FE method is a numerical method used to solve differential equations arising in engineering and scientific modeling by dividing the model into smaller and simpler finite elements. The method is widely used for analysis in mechanical engineering and has recently entered common use in molecular biology [17,18,19,20]. We then determined the risk factors affecting the severity of placental abruption by changing collision velocity, penetration depth in the abdomen, and diameter of the steering wheel.

### 2.1. FE Model of a Pregnant Woman

**Figure 1:**
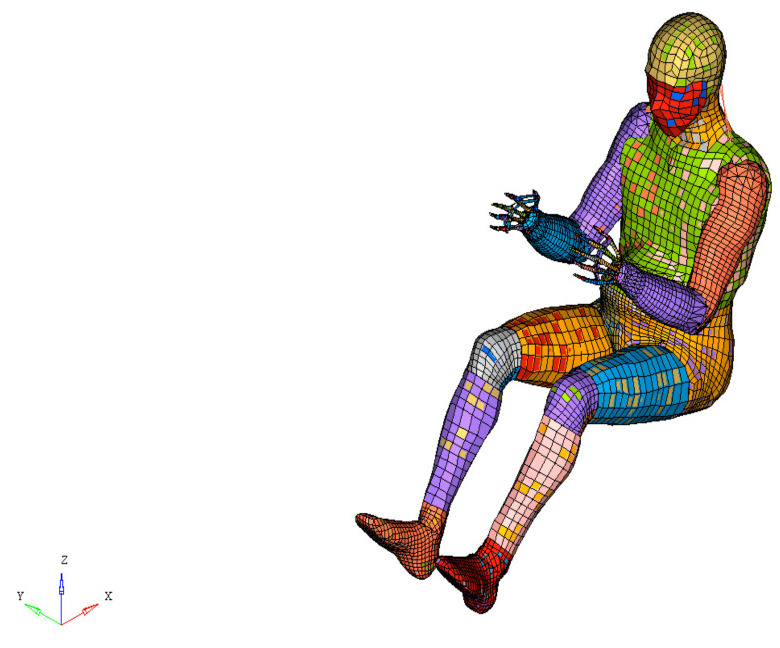
Finite element (FE) model of a pregnant woman.

The height of the model of the pregnant woman was set at 153 cm, which is a common height for pregnant women in Asian countries and corresponds to the fifth percentile of the height of American women (AF05). This also allowed us to easily compare the results with those of previously reported experiments using dummy models of pregnant women of AF05 size. The placenta was located in the uterine fundus, which is the most common location of the placenta. The gestational age of 30 weeks was similar to the condition of dummy models of pregnant women reported previously. The FE model is illustrated in Figure 1. There were 83,149 nodes and 192,349 elements in the uterine model, including the uterine model described thereafter.

**Figure 2:**
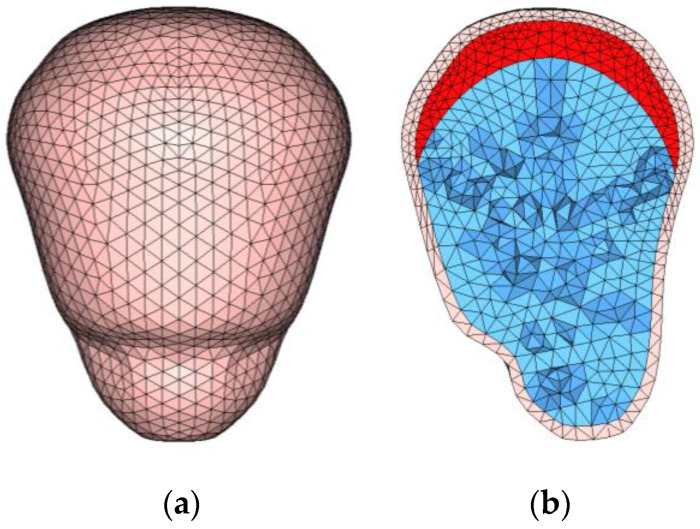
FE uterine model and its sagittal section. (**a**) overview of uterine FE model, (**b**) sagittal section of uterine FE model.

The uterine model was developed in three steps. We first chose a median United States adult male (AM50) FE model in THUMS ver. 1.61 and scaled it to the AF05 size as a whole-body FE base model. We then constructed FE models of the uterus, placenta, and amniotic fluid as shown in Figure 2 and inserted them into the abdomen of the base model. We finally inflated the uterine model to a 30-week gestation size within the whole-body model. When constructing the FE model, we used a commercial modeling tool, Hyperworks, by Altair (Engineering Inc., Troy, MI, USA).

The uterine model refers to the anthropometry of a pregnant female determined from the computed tomography data published by Loftis et al. [27]. For simplicity, we assumed that the uterine wall thickness was an even 7 mm in the uterine model. There were 9549 nodes and 58,223 elements in the model. The dynamic characteristics of the uterus, placenta, and amniotic fluid model refer to published data [28,29,30]. The uterus, placenta, and amniotic fluid were modeled as elastic bodies, and their density, Young’s modulus, and Poisson coefficient were configured as shown in Table 1.

### 2.2. Impact Simulation

For simulation of the impact in the collision, a numerical simulation was conducted using the developed model. For analysis of the area of placental abruption, a rigid impactor imitating the lower rim of the steering wheel was set up in front of the FE model of a seated pregnant woman; it approached the woman model horizontally to press against the abdomen and continued to move until reaching the endpoint at a depth of 90 mm.

We ran numerical simulations in which we varied the diameter of the impactor, the impact position, and the impact velocity. Three different sizes of impactor, with diameters of 25, 50, and 100 mm, penetrated the abdomen. The impact positions were the midline of the umbilicus and 30 mm above and below the umbilicus. All these levels were lower than the xiphoid process and higher than the ileum. The impact velocities of the impactor were 3 m/s (10.8 km/h), 6 m/s (21.6 km/h), and 9 m/s (32.4 km/h).

In the simulation of the impactor penetration, shear stress along the utero–placental interface (UPI) was analyzed for each element; a failure criterion of the UPI of 15.6 kPa was taken from the literature [31]. UPI elements subjected to shear stress over the failure criterion were assumed to undergo placental abruption.

The area of placental abruption was approximated as the number of elements in which the shear stress exceeded the failure criterion because the elements in the uterine FE model were mostly uniform in area. We estimated the percentage fatality of fetuses from the simulated area of placental abruption, referring to data of the correlation between the placental abruption area and fatality in real-world MVCs [24]. Placental abruption was classified into three grades of the percentage of separated area, i.e., less than 30% (grade 1), from 30% to 50% (grade 2), and more than 50% (grade 3). The corresponding percentages of fetal fatalities after vaginal delivery were 30%, 79%, and 100%, respectively [24].

## 3. Results

### 3.1. Validation of the Model

**Figure 3:**
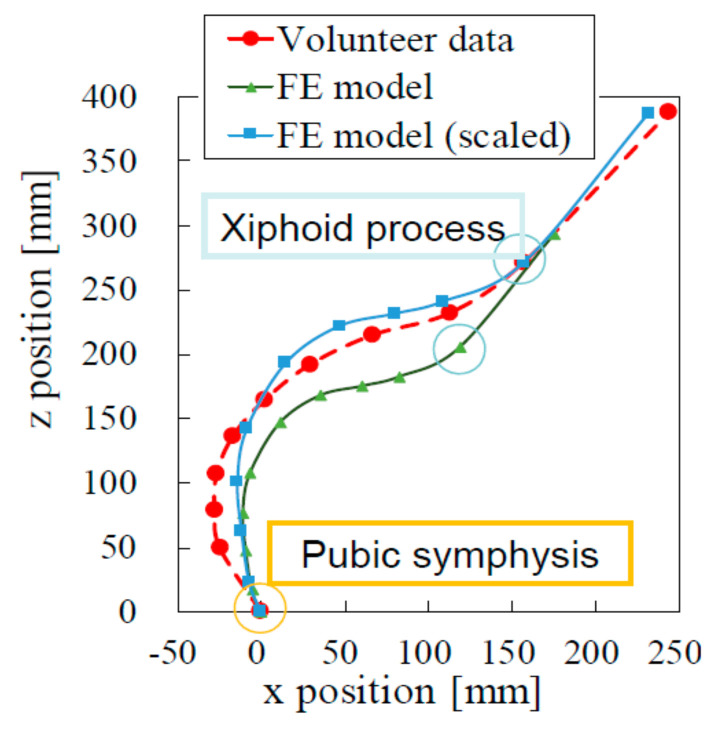
Abdominal curves of the FE model of the pregnant woman and of volunteer data. The curves plotted with triangles, squares, and circles correspond, respectively, to the original FE model, the scaled FE model, and volunteers.

For anthropometric validation, we fitted two abdominal curves, one indicating the sagittal section of the pregnant FE model and the other indicating data of pregnant volunteers published in the literature [32] (Figure 3). As the steering wheel comes closer to the abdomen of the pregnant driver, it is more likely to damage a pregnant woman’s abdomen and her fetus. It was thus important to validate the body shape of our FE pregnant model with the body shapes of real volunteer pregnant women, as shown in Figure 3. Following the height difference between the model and the average of volunteers, we scaled the pregnant FE model so that both the xiphoid process and the pubic symphysis were located at the same x- and z-coordinates, as illustrated in Figure 3. The abdominal curves of the scaled FE model and the volunteer data matched well. We therefore concluded that the FE model was anthropometrically valid.

**Figure 4:**
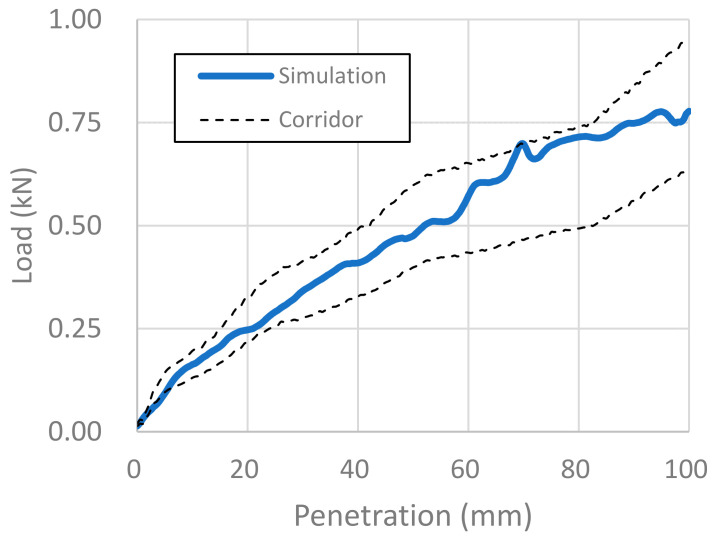
Biomechanical validation of the impactor penetration at a collision velocity of 3 m/s.

**Figure 5:**
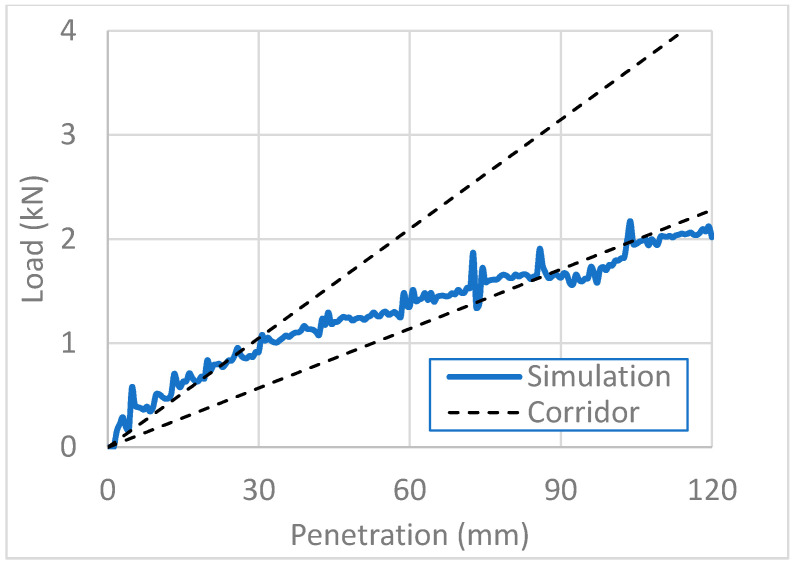
Biomechanical validation of the impactor penetration at a collision velocity of 6 m/s.

**Figure 6:**
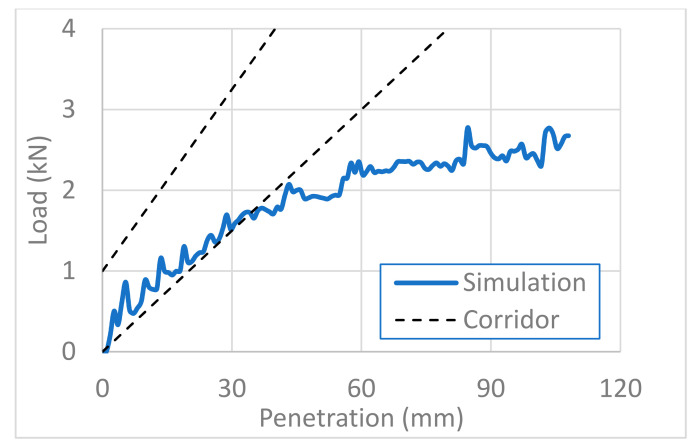
Biomedical validation of the impactor penetration at a collision velocity of 9 m/s.

For biomechanical validation, we compared the abdominal impact response of the FE model of the pregnant woman with that of a post-mortem human subject (PMHS) published in the literature [33]. It was important to fit well the physical behavior of our FE pregnant model with that of the real pregnant women’s body. The FE model, however, has unavoidably limited accuracy in reproducing real physical behaviors. We thus validated the FE model biomechanically by comparing it with PMHS data for a real body in Figure 4, Figure 5 and Figure 6. We ranged corridors in 10% higher and lower than the PMHS responses at collision velocities of 3, 6, and 9 m/s. The pregnant FE model responses were compared with the corridors for each collision velocity in Figure 4, Figure 5 and Figure 6, where the x-axis and y-axis are, respectively, the penetration depth of the impactor and the load imparted on the abdomen by the impactor. For collision velocities of 3 and 6 m/s, the FE model response fitted the corridor well at all depths in our simulation, while it retracted from the corridor at penetration depths greater than 35 mm at a collision velocity of 9 m/s. We thus concluded that our FE model of a pregnant woman is valid for the simulation of impactor penetration at collision velocities of 3 and 6 m/s.

### 3.2. Placental Abruption Area

**Figure 7:**
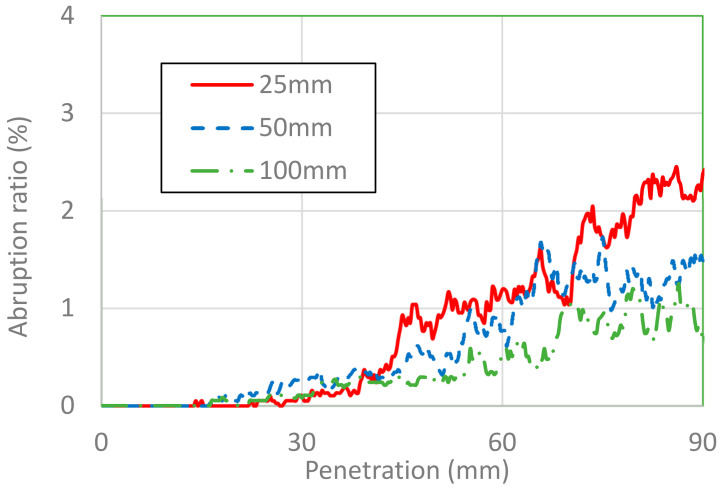
Placental abruption area versus penetration depth for impactor diameters of 25, 50, and 100 mm at an impactor velocity of 3 m/s.

**Figure 8:**
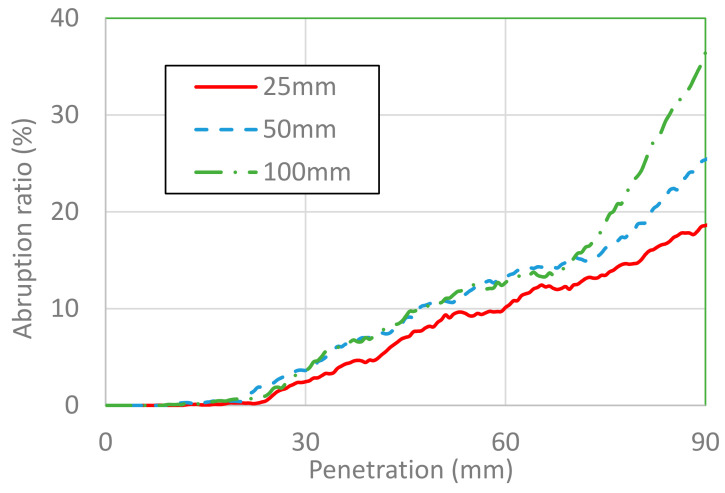
Placental abruption area versus penetration depth for impactor diameters of 25, 50, and 100 mm at an impactor velocity of 6 m/s.

**Figure 9:**
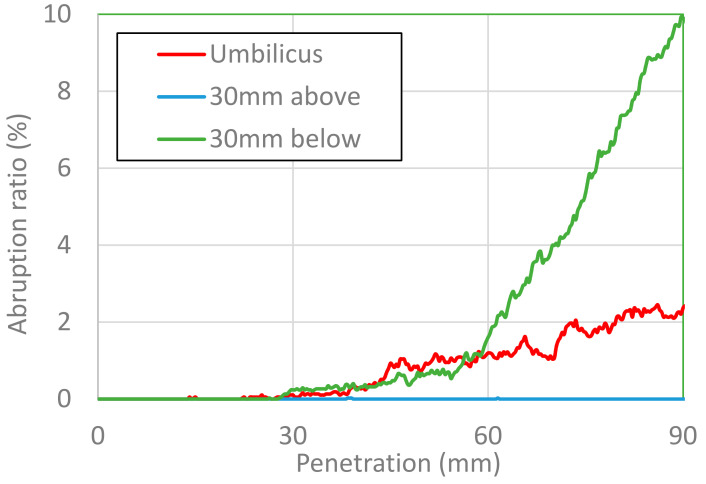
Placental abruption area versus the depth of penetration at the umbilicus, 30 mm above it, and 30 mm below it at an impactor velocity of 3 m/s.

**Figure 10:**
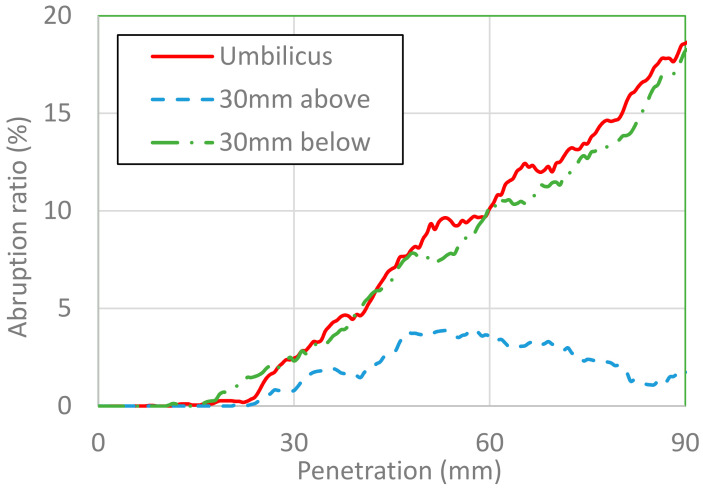
Placental abruption area versus depth of penetration at the umbilicus, 30 mm above it, and 30 mm below it at an impactor velocity of 6 m/s.

The area of placental abruption is expressed as the area of the separated placenta in percentage. Figure 7 and Figure 8 show the area of placental abruption at impactor velocities of 3 and 6 m/s, respectively, for different impactor diameters. In Figure 7, Figure 8, Figure 9 and Figure 10, the x-axis and y-axis are, respectively, the penetration depth of the impactor and the placental abruption area. These figures depict the progression of placental abruption in accordance with impactor penetration. For all combinations of impactor velocity and diameter, the area of placental abruption increased as the impactor more deeply penetrated the abdomen of the FE model of the pregnant woman.

At an impactor velocity of 3 m/s, the area of placental abruption was approximately 2.5% at most for an impactor diameter of 25 mm. This abruption was classified as grade 1, for which the percentage of fatality of fetuses is 30%. At a velocity of 6 m/s, the placental abruption area was approximately 35% at most for an impactor diameter of 100 mm. This abruption was classified as grade 2, for which the percentage of fatality of fetuses is 79%. At both impactor velocities, there were no marked differences in the abruption ratio in relation to the impactor diameter until a penetration depth of approximately 70 mm.

Figure 9 and Figure 10 show the placental abruption areas versus the penetration depth for a variety of impact positions at impactor velocities of 3 and 6 m/s, respectively. The diameter of the impactor was set at 25 mm.

At an impactor velocity of 3 m/s, no placental abruption was observed when the impactor penetrated 30 mm above the umbilicus. When the impactor penetrated 30 mm below the umbilicus, the placental abruption area markedly increased for a penetration depth greater than 60 mm and finally reached 10%, which is four times that of the penetration at the umbilicus level. These abruption areas were classified as grade 1.

At an impactor velocity of 6 m/s, the placental abruption percentage increased when the impactor penetrated more than 20 mm and finally reached approximately 20% when the impactor penetrated at the umbilicus and 30 mm below the umbilicus, whereas it was approximately 4% when the impactor penetrated 30 mm above the umbilicus. All these abruption areas were classified as grade 1.

## 4. Discussion

Using our novel FE model, areas of placental abruption can be investigated, and fetal outcome precisely evaluated for different collision situations. Previously, because placental abruption typically occurs when pregnant women are severely injured in MVCs, it was not evaluated in case of minor injuries, such as bruising, lacerations, and contusions, in the event of an MVC [16]. As, in reality, placental abruption has occurred in mildly injured pregnant women following a collision [16], a biomechanically relatively minor deformation force is sufficient to shear the placental attachments away from the decidua basalis. The prognosis of the fetus depends on the proportion of the separated area, and we thus created our model on this basis [24]. In the clinical field, the proportion of the separated area of the placenta has been estimated in retrospect and does not serve as a guide to therapy, and the severity of the disease has been expressed in terms of clinically recognized factors, such as degree of hypotension, disseminated fibrin embolisms, and in vivo defibrination of the blood leading to uncontrollable hemorrhage [24]. Therefore, considering the area of separation with our model provides indices of the severity of placental abruption and fetal fatality due to trauma.

The results of this study suggest that the area of placental abruption increases nearly 10-fold when the impactor collision velocity doubles. At both collision velocities of 3 and 6 m/s, the percentage abruption area increased with the penetration depth. This suggests that a high collision velocity has negative outcomes for a pregnant woman and her fetus. The impactor velocity of 6 m/s corresponds to 21.6 km/h (13.5 mph), which is below the speed limit of a general road. Therefore, unrestrained pregnant drivers possibly suffer from fatal placental abruption and fetus loss even in collisions at low vehicle speed, when the maternal body slips freely on the driver’s seat to the lower rim of the steering wheel. This result agrees well with the results of previous real-world collision analyses and sled tests using a pregnant dummy model [14,15]. Furthermore, the present results show the risk of suffering placental abruption quantitatively, which has not been reported previously. The highest priority is therefore to recommend a correct seatbelt use to avoid contact with the steering wheel. However, if the seating position of the driver is near the steering wheel, some pregnant women cannot avoid contact of the steering wheel with the abdomen. Furthermore, in a high-velocity collision, forces of certain magnitudes act on the body through the penetration of the seatbelt [34]. Therefore, reducing the collision velocity contributes to better outcomes of the pregnant driver and fetus in vehicle collisions. Even if a vehicle is unavoidably involved in a frontal or rear collision, a collision avoidance system will help reduce the collision velocity and the chance of fetus loss. This preventive measure based on the present results agrees well with a measure suggested by analyses of a national collision database involving pregnant women [11].

The results of this study also reveal that the placental abruption area was smaller after penetration 30 mm above the umbilicus than after penetration at the umbilicus or below it. However, in this situation, upper abdominal organs such as the liver might be severely deformed rather than the uterus. The deformation shown in the present results may correspond to severe liver damage or hepatorrhexis in real collisions, possibly leading to fatal hemorrhage and fetus loss [35,36,37]. Whether the penetration is higher or lower than the umbilicus, the application of an external force between the xiphoid process and the ileum may affect both maternal and fetal outcomes.

This study has two limitations. First, the FE model of the pregnant woman does not perfectly reflect a real pregnant human body, particularly in terms of the anthropometry of the pelvis, because it was built by scaling an FE model of a non-pregnant person. Second, in this study, the placenta was located only at the uterine fundus and not at the frontal, rear, or lateral uterine walls. In future work, it will be important to evaluate placental abruption areas for a variety of locations of the placenta. The present study simulated MVCs in which the steering wheel directly impacted the abdomen of a pregnant woman without any restraint by a seat belt. Further study is required to reveal how much safety equipment, including an airbag, reduces or increases placental abruption.

In this study, because the severity of placental abruption depends on the area of separation, we developed a novel mathematical model for quantitatively evaluating the separated area in placental abruption. This study provides the first simulation results to determine the area of placental abruption following the penetration of the steering wheel into the abdomen. Subsequently, the factors that promote placental abruption in MVCs were identified. Further simulations based on this study will clarify the conditions that likely contribute to fetus losses and the factors that may prevent severe outcomes for pregnant women and fetuses.

## 5. Conclusions

This paper simulated placental abruption following penetration by the steering wheel in an MVC using an FE model of a pregnant woman in late term. As the collision speed increased from 3 to 6 m/s, the area of the abrupted placenta increased 10-fold. This result implies that even low-speed vehicle collisions cause negative maternal and fetal outcomes owing to the abruption of the placenta if the pregnant driver is not restrained. Additionally, the simulation showed that impactor penetration 30 mm below the umbilicus resulted in a larger area of abruption than impactor penetration at the umbilicus when the placenta was located in the uterine fundus. This study is the first to estimate the placental abruption area by computing stress along the UPI and comparing it with failure criteria. The results support that a reduction in the collision speed and a seatbelt restraint at a suitable position are important to protect pregnant women and their fetuses in an MVC.

## Figures and Tables

**Table 1:** Dynamic characteristic parameters of the uterine model.

Tissues	Specific Gravity (kg/m^3^)	Young’s Modulus (kPa)	Poisson Coefficient
**Uterine wall**	1006	467	0.49
**Placenta**	1006	38	0.49
**Amniotic fluid**	1000	2	0.49

## Data Availability

The data presented in this study are available upon request from the corresponding author.
